# Supplementary material for: First cases of SARS-CoV-2 infection and secondary transmission in Kisumu, Kenya
Source: PLOS Glob Public Health. 2022 Sep 8;2(9):e0000951. doi: 10.1371/journal.pgph.0000951 (PMC10021778; doi:10.1371/journal.pgph.0000951)
Supplement: Attachment — Submitted filename: Response to Reviewers.docx [file pgph.0000951.s003.docx]

**Response to reviewers**

PGPH-D-21-00645
First cases of SARS-CoV-2 infection and secondary transmission in Kisumu, Kenya
PLOS Global Public Health

Dear Sir/Madam –

Thank you for your review of our manuscript. Please find our responses below and the changes in the attached revised draft.

Sincerely,
Beth A Tippett Barr, DrPH

Journal Requirements:

1. Please provide additional details regarding participant consent. In the ethics statement, please ensure you have specified what type you obtained (for instance, written or verbal, and if verbal, how it was documented and witnessed).

- Thank you. We have added these details into the ethics statement, which now reads: “All participants aged 18 and above voluntarily provided written consent for themselves (ages 18+), witnessed by the study data collector and Ministry of Health outbreak response personnel present. Children aged 13-17 provided written assent in addition to written parental consent.”

2.  Please provide separate figure files in .tif or .eps format only, and remove any figures embedded in your manuscript file.  If you are using LaTeX, you do not need to remove embedded figures.

For more information about figure files please see our guidelines: 
<https://journals.plos.org/globalpublichealth/s/figures>

- Thank you – we have done as advised

 3. In the online submission form, you indicated that "Data can be provided by the lead author upon request."

- We have uploaded the data in Excel format as supplementary information.

4. Please amend your detailed Financial Disclosure statement. This is published with the article, therefore should be completed in full sentences and contain the exact wording you wish to be published.

i). State the initials, alongside each funding source, of each author to receive each grant.

ii). State what role the funders took in the study. If the funders had no role in your study, please state: “The funders had no role in study design, data collection and analysis, decision to publish, or preparation of the manuscript.”

- Thank you – the revised statement now reads: “This investigation was funded through a grant from the CDC Foundation to the University of California San Francisco (PMM, JLK, CRC). The opinions expressed by authors contributing to this manuscript do not necessarily reflect the opinions of the CDC Foundation, the U.S. Centers for Disease Control and Prevention, or the institutions with which the authors are affiliated. The funders had no role in study design, data collection and analysis, decision to publish, or preparation of the manuscript.”

Additional Editor Comments (if provided):

Thank you very much for your submission. Your work entitled ""First cases of SARS-CoV-2 infection and secondary transmission in Kisumu, Kenya" explores precisely the subject matter we hope PLOS Global Health will be able to focus on. The experience of early cases of COVID in Kisumu is very important for our expanding understanding of COVID in different contexts but also the use of FFX may be particularly important looking forward to future pathogens of interest. You will see the reviewers appreciated the subject and the work done here, but would like to have further clarifications in order to publish the paper, particularly involving the communication of the statistics used. Some details can be easily clarified; some may not be and that is understandable. Further figures/graphs to better communicate the concepts were also suggested. I hope this is helpful. This work is very important and I hope we are able to publish this paper.

- Thank you for the additional context you have provided with the reviewer comments. We have endeavored to meet all the requests but do want to acknowledge that the request for ‘more advanced statistics’ was a challenge. This study was not intended to be a complex research study but rather a rapid epidemiological assessment of an emerging disease. I have added some language in the methods about the use of the universal “WHO Unity Study” protocol on emerging pathogens and explained that the small sample size with proportionally small severe outcomes limits our ability to conduct complex statistical techniques.
- I have embedded the only other two papers from Africa that I can find which have utilized this same WHO Unity protocol for a rapid assessment of COVID-19 in their context – please see the simple descriptive approach taken by each, similar to what we have included in this paper.

**Comments to the Author**

1. Does this manuscript meet PLOS Global Public Health’s [publication criteria](https://journals.plos.org/globalpublichealth/s/criteria-for-publication)? Is the manuscript technically sound, and do the data support the conclusions? The manuscript must describe methodologically and ethically rigorous research with conclusions that are appropriately drawn based on the data presented.

Reviewer #1: Yes

Reviewer #2: Partly

2. Has the statistical analysis been performed appropriately and rigorously?

Reviewer #1: No

Reviewer #2: No

- Thank you – please see our response to your specific comments on the statistical methods below.

3. Have the authors made all data underlying the findings in their manuscript fully available (please refer to the Data Availability Statement at the start of the manuscript PDF file)?

The [PLOS Data policy](https://journals.plos.org/globalpublichealth/s/data-availability) requires authors to make all data underlying the findings described in their manuscript fully available without restriction, with rare exception. The data should be provided as part of the manuscript or its supporting information, or deposited to a public repository. For example, in addition to summary statistics, the data points behind means, medians and variance measures should be available. If there are restrictions on publicly sharing data—e.g. participant privacy or use of data from a third party—those must be specified.

Reviewer #1: Yes

Reviewer #2: Yes

4. Is the manuscript presented in an intelligible fashion and written in standard English?

PLOS Global Public Health does not copyedit accepted manuscripts, so the language in submitted articles must be clear, correct, and unambiguous. Any typographical or grammatical errors should be corrected at revision, so please note any specific errors here.

Reviewer #1: Yes

Reviewer #2: Yes

5. Review Comments to the Author

Please use the space provided to explain your answers to the questions above. You may also include additional comments for the author, including concerns about dual publication, research ethics, or publication ethics. (Please upload your review as an attachment if it exceeds 20,000 characters)

Reviewer #1: The manuscript seems to interesting but overall analysis is very poor, basic descriptive statistics no test statistics in table. In addition, the relevance of paper is not clearly out and no recommendations etc

I would suggest to re-do analysis with advance analysis not only descriptive.

- Thank you for this suggestion. I would like to acknowledge that the request for ‘more advanced statistics’ was a challenge and is something the authors discussed at length before writing the paper. This study was not intended to be a complex research study but rather a rapid epidemiological assessment of an emerging disease. We also consulted again with a third statistician who confirmed that the use of complex analysis techniques with this small sample size would be inappropriate and that any regression model would not converge.
- I have added some language in the methods section about the use of the universal “WHO Unity Study” protocol on emerging pathogens and explained that the small sample size with proportionally small severe outcomes limits our ability to conduct complex statistical techniques.
- I have embedded below (and referenced in the revised paper) the only other two papers from Africa which have utilized this same WHO unity protocol for the rapid assessment of COVID-19 initial outbreaks – please see the simple descriptive approach taken by each, similar to what we have included in this paper.

Reviewer #2: The authors described an investigations they conducted among the first 152 cases of laboratory confirmed SARS-CoV-2 cases in Kenya. The generally could provide useful information to public health practitioners and policy makers on the transmission cycle in some African countries. The manuscript however lack clarity in coherence and presentation of thoughts hence making it difficult to read and related the events presented.

- Thank you for this observation. We have revised the results and discussion sections to address your concerns on coherence.

My comments are below:

Abstract
The abstract needs some additional details to bring out the findings of the work. Eg. Authors could highlight the nature of the 152 confirmed cases? How many were primary cases? How many secondary cases? Of the 248 contacts, how many were household contacts or non-household contacts. Authors should rewrite the abstract in a more clearer way.

- Thank you – we have revised the abstract, particularly on the results and discussion

Line 40: Authors should correct the statement -- "This study, conducted June 10–October 8, 2020, included".

- Thank you for this comment, but I’m not sure what you mean by ‘correct the statement’ – it’s grammatically correct and the dates are correct. Thank you

Line 46: The are double brackets in the text. Authors should remove this.

- Thank you – corrected.

Results
The authors should mention the breakdown of the 152 laboratory confirmed cases. How many of the primary cases were identified through the private laboratories?

- Approximately 10% of cases were identified by private laboratories; I have revised the wording in the methods section to clarify this; please note that all diagnoses were confirmed by the study laboratory.

Line 106: Definition of close contact is a bit confusing? How were authors able to ascertain the 2 meters close contacts with primary cases? Did they measure this?

- Per the methods section (now line 119), this was self-reported.

What was the duration of contact between primary cases and contacts?

- Time clarified to >15mins

Were the cases of contact not observing any of the COVID-19 protocols? Was any of them in mask? What was the national COVID-19 regulations at the time of the study?

- Thank you – I have clarified this in the methods section. Mask mandates were not yet in place at the time of this study. The only participants who can be assumed to be wearing masks at any time are health care workers in the workplace.

Line 113: Authors should include the number of patients tested and those positive or negative for the cases and contact groups? This will help bring some clarity to the follow up done and those who turn out positive.

- Thank you for this comment. For the cases, as described in the methods section, recruitment was done from cases identified as positive in the laboratory, which at the time was conducting testing for the national Ministry of Health. We do not have data on negatives tested during this time, and as there was no clear testing strategy in place in this early stage of the pandemic, this detail would not provide much additional context to the picture as the denominator would be meaningless.
- For the contact group, we describe in the methods section how frequently testing was conducted, and in the results section how many were subsequently infected. We have also added a new table describing factors associated with COVID-19 acquisition which were specific to cases vs contacts.

Line 116- 120: What were the whole and serum blood samples collected used for? What laboratory analysis was performed on them? What were the immunological parameter measured? What was the clotted blood used for?

- Thank you for this comment. We have recently received the final results from the testing that was conducted using those methods and have included them in the results section.

Line 143: Authors definition of severe disease is also not clear?? Was this based on a national guideline? How did authors distinguish between moderate and severe disease? How come death was considered as severe disease?

- Thank you for this comment. In the ‘statistical analysis’ section of the methods we provide the following: “Severe disease was defined as requiring hospitalization or resulting in death”. The lack of standardized reporting tools in this context limited our ability to categorize severity of illness other than by self-reported factors including hospitalization, ICU and next-of-kin-reported death.

Line 159: Table 1 should be reconstructed to describe the participant characteristics by cases and contact without inclusion of gender comparison. Including gender at this stage makes it difficult to read through the results. Table 1 is also not referenced in the text. The subtotal indicated in the table should also be removed. All the levels of age, occupation and activities have the same sub-total so it not clear why authors kept repeating this.

- Thank you for this comment. We prefer to keep the breakdown of cases and contacts by sex. As you may remember, early in the pandemic there was a lot of discussion around the different experiences of men vs women with COVID-19, and the disaggregation by sex in Table 1 reflects this in our context. It also clearly shows how the disease was male/occupation driven at the very beginning.
- Table 1 is referenced at the end of the first sentence in the results. We can move it elsewhere if you feel it could be better positioned.
- We have removed the sub-totals as advised.

Line 166: Figure 2 intends to describe the trends among cases. Authors could best represent this as line plots by dates. Its difficult to understand the rising cases over time for the different occupational groups.

- Thank you for this observation. We have changed the figure to a line plot as requested.

Line 179: Authors mentioned about combination of symptoms but its not clear which symptoms they were talking about?

- Thank you – I have clarified “any symptoms”

Line 191: Table 3: What are the various definitions for the different comorbidities mentioned?

- Per the methods section, all comorbidities are self-reported. I have added ‘self-reported’ in this line as well to be clear.

Line 201: Table 4 seem to lack clarity? What is the odd ratio measuring? Is it the risk of hospitalisation or death? This is quite confusing and ambiguous. Authors should look at this separately as either risk of hospitalisation separately from deaths. Again doing this analysis for particular age categories is also difficult to interpret along the other variables?

- Thank you for pointing this out. We have restructured this table (now Table 3, as the original Tables 2 and 3 were merged) to clarify this is the risk of hospitalization vs no hospitalization for each comorbidity. We have removed age groups and added ORs and p-values.

Line 220: Line 220: Authors seem to show some risk factor analysis being presented but interpretation is not clear?

- Thank you – we have added a new table (Table 4) showing this analysis, and we hope the narrative is subsequently more clear as well.

"those with known comorbidities were no more likely to acquire COVID-19 than those without comorbidities (OR= 1.46 [95%CI 0.42- 5.12], p=0.56)" The odd ratios are all not significant and their interpretation is not relevant not relevant in explaining the risk of infection? Authors should conduct proper risk factor analysis to clearly show the risk of infection for the contacts groups.

- Thank you – we have now referenced the new table added to the results section (Table 4).

Line 235: Authors mentioned secondary transmission occurred in 27 contacts were identified by 12 cases??? I did not see this clearly in the results. Authors should relook at the statement and describe this well.

- Thank you – the transmission dynamics is a subsection in the results section. All cases are included in the analysis, but of those 152 total cases, 27 were secondary transmissions from 12 of our 125 primary cases.
- I have also added this statement into the first sentence of the results (line 173) so that it’s more clear from the start of the results.

Line 235: Again this particular statement was not significant so its not clear why authors still considered the finding as relevant in their discussion.

- Thank you for this observation. We have removed this sentence.

Line 237: Authors did not state whether the contacts or primary cases identified were in mask or used any of the COVID-19 prevention protocols? Is it that the transmission among contracts of health workers was zero because they were in mask?

- Thank you – I have added a sentence to clarify the social context at the time.

Authors need to get assistance of epidemiologist and biostatisticians to help them reorganize their data in a way that will be more meaning to readers.

- Thank you for this suggestion. We had 2 statisticians and 4 PhD Epi’s in the co-author group. Many analysis limitations were in place by default based on the study design (rapid assessment with a small ‘n’). We consulted an additional statistician and reorganized some tables and narrative; we hope this will meet with your satisfaction
