# Supplementary material for: First cases of SARS-CoV-2 infection and secondary transmission in Kisumu, Kenya
Source: PLOS Glob Public Health. 2022 Sep 8;2(9):e0000951. doi: 10.1371/journal.pgph.0000951 (PMC10021778; doi:10.1371/journal.pgph.0000951)
Supplement: Attachment — Submitted filename: Response to reviewers.docx [file pgph.0000951.s004.docx]

**Response to reviewers**

PGPH-D-21-00645
First cases of SARS-CoV-2 infection and secondary transmission in Kisumu, Kenya
PLOS Global Public Health

Dear Sir/Madam –

Thank you for your second review of our manuscript. Please find our responses below and the changes in the attached revised draft.

Sincerely,
Beth A Tippett Barr, DrPH

Journal requirements:

1. Please review your reference list to ensure that it is complete and correct. If you have cited papers that have been retracted, please include the rationale for doing so in the manuscript text, or remove these references and replace them with relevant current references. Any changes to the reference list should be mentioned in the rebuttal letter that accompanies your revised manuscript. If you need to cite a retracted article, indicate the article’s retracted status in the References list and also include a citation and full reference for the retraction notice.
   - Thank you for this instruction. We have done as requested. One reference was removed based on other edits required from reviewers, and one was added that had inadvertently been dropped from previous versions. I have also confirmed that none of the cited articles have been listed as retracted or removed on pubmed.

Comments to the Author:

1. Reviewer #2: Authors have addressed most of the comments raised. I do understand there might be limitations in the nature of data acquired during the early stages of the pandemic but nevertheless, this work provides some information that may be useful to the general public.
   - Thank you for your observation; we appreciate the support
2. Reviewer #3: Line 94 - is there a reference to support this information?
   - I have reorganized the sentence to clarify that the data all falls under reference number 9; the reference list was also corrected so that number 9 refers to the correct document.
3. Line 127 - you say mask mandates were not implemented, but in the rest of the manuscript, you say that wearing of masks in public was mandatory... please align all comments on mask wearing to give same meaning and interpretation
   - Thank you for catching this discrepancy. I have revised the statements so that it’s clear there were no public mask mandates at the beginning of the study, but that they became a mandate during study implementation period.
4. Discussion - has much of results presented in it and reads somewhat like the results section. Review discussion to present more of authors thoughts on the study findings and what next steps should look like or authors thoughts on follow up studies
   - Thank you for this observation – I have revised the discussion section to read more like discussion than results, including further points on why the discussion points raised are important to consider for future outbreak response. I have also added a conclusion section summarizing thoughts and relevance to future outbreaks as well as the ongoing COVID-19 pandemic.
